# Heavy spenders in digital games: expenditure disparities and associations between microtransactions, gaming disorder and gambling disorder among adolescents

**DOI:** 10.3389/fpubh.2026.1867460

**Published:** 2026-08-21

**Authors:** Markus Meschik, Elena Hammer, Doris Malischnig, Natalia Wächter, Mark D. Griffiths

**Affiliations:** 1 Department of Social Sciences, University of Applied Sciences, St. Pölten, Austria; 2 Department Building, Environment and Society, FH JOANNEUM, Graz, Austria; 3 Therapy Center Ybbs, Vienna Healthcare Group, Ybbs an der Donau, Austria; 4 Department for Educational Sciences, University of Graz, Graz, Austria; 5 Psychology Department, Nottingham Trent University, Nottingham, United Kingdom

**Keywords:** adolescents, free-to-play, gambling disorder, gaming disorder, in-game purchases, loot boxes, microtransactions

## Abstract

Free-to-play games have transformed the economics of digital play, shifting revenue from upfront sales to continuous microtransactions. Research on the free-to-play model has largely focused on specific mechanics such as loot boxes or pay-to-win systems. However, little is known about how adolescents’ spending behaviour relates to problematic engagement. Drawing on survey data from 2,308 Austrian students aged 10–19 years (48.8% male; M = 14.3 years, SD = 2.1), the present study examined the distribution of in-game expenditure and its association with gaming disorder, gambling disorder, and socioeconomic status. Expenditure was highly unequal: 10% of players accounted for 61.4% of all spending, and heavy spenders showed higher prevalences of gaming disorder and gambling disorder than casual spenders. Expenditures were not significantly associated with socioeconomic status, suggesting that high spending is not limited to adolescents with greater family affluence. The concentration of spending resembles patterns observed in other highly unequal expenditure markets, including gambling, suggesting potential structural similarities between the two industries. These findings raise questions about the assumption that microtransactions are harmless optional features and suggest that policy efforts targeting only loot boxes may not fully address potential risks associated with broader monetization practices among adolescent gamers.

## 1 Introduction

The digital games industry has increasingly moved away from selling games as standalone products towards offering them as ongoing services. This shift, embodied by the free-to-play model, relies on in-game transactions rather than upfront purchase prices to generate revenue. The rising revenue of the gaming industry highlights the success of this model. In 2025, more than 55% of the industry’s $188.8 billion revenue came from mobile games, which are mostly financed through in-game purchases (1). In-game purchases may include purely cosmetic content, such as skins or costumes that do not affect gameplay, or items that confer gameplay advantages, a practice commonly referred to as pay-to-win. Such content can be acquired either through direct purchase or via randomised mechanisms such as loot boxes, where the specific item obtained is unknown at the time of purchase.

Children and adolescents constitute a core demographic of digital game players, a fact also reflected in their engagement with in-game purchases. Given the substantial revenues generated by this industry, concerns have been raised about how spending habits develop within this young audience. In particular, it remains unclear whether their purchases typically remain within age-appropriate allowance boundaries or whether this financially and developmentally vulnerable group is prone to systematic overspending.

### 1.1 Extent of in-game purchases among children and adolescents

Earlier surveys provide an initial indication of how widespread in-game purchasing actually is among minors. Over the course of a year, between 28% (2) and 36% (3, 4) of adolescents spent money on in-game purchases, with male adolescents from low educational backgrounds being the primary consumers (2, 5). Spending money in games appears to be common among children and adolescents, although there is limited research on the amounts of money spent and the impacts compared to adult gamers, where factors such as a younger age, male gender, and the presence of gambling problems appear to contribute to higher expenditure (6).

Moreover, recent surveys differentiate between various forms of in-game purchases. Buyers in pay-to-win games are primarily male and generally younger individuals with lower educational backgrounds (7). Among adolescents, the purchase of loot boxes is particularly popular. Surveys in England found that 40% of adolescents reported buying loot boxes in the past month (8, 9). Other surveys reported slightly lower figures of 27% (10) and 29% (11) with a higher prevalence of gaming and gambling disorders among buyers. Overall, the current body of research on adolescent players provides only limited insights into the spending patterns among children and adolescents, highlighting the need for further empirical work in this age group.

The shift towards a service-based revenue model in the games industry has intensified efforts to maintain player engagement over time. In doing so, developers have increasingly drawn inspiration from the gambling industry, leading to a growing convergence between gaming and gambling practices. This overlap is evident not only in social or casino-style games, which directly emulate gambling mechanics (12), but also across a wide range of in-game purchase systems. Among these, loot boxes have received the most academic attention. Loot boxes dispense pseudo-randomised rewards and have been shown to mirror key characteristics of gambling (8, 13). Empirical studies have established associations between loot box engagement and problematic gambling behaviours among both adult players (6, 8, 14–19) and adolescent players (20). Beyond associations with gambling-related harm, several studies have also found positive associations between loot box engagement and indicators of gaming disorder (19, 21, 22).

### 1.2 Key factors in problematic behaviour

To understand players’ spending behaviour and the design decisions that encourage it, cognitive bias theory (23) provides a useful framework, suggesting that individuals systematically deviate from rational decision-making due to predictable mental shortcuts and distortions in judgement. Cognitive biases such as the sunk-cost fallacy, the illusion of control, and the near-miss effect are well established in gambling research (24–27) and may similarly influence players’ willingness to spend on in-game microtransactions. These biases lead individuals to overestimate potential rewards, underestimate losses, and persist in spending behaviour even when rational evaluation would suggest stopping. From this perspective, heavy in-game spending reflects not only economic decision-making but may also result from cognitive biases triggered or reinforced by a specific game design such as artificial scarcity (27).

Loot boxes have been associated with gambling-related mechanisms such as near misses (8, 25), physiological arousal during openings (28), and cognitive biases including the sunk cost fallacy (29). Because they rely on randomised reward schedules, loot boxes reinforce continued engagement even when the desired item is not obtained. Similar reinforcement effects have been observed in gambling, where near misses are known to increase persistence and emotional arousal (30).

Moreover, loot boxes can be purchased and opened repeatedly, increasing event frequency, the rate at which reward-related events occur. Systematic reviews have identified event frequency as a key factor linking microtransactions to problematic engagement and gambling-like behaviours (31). This mirrors findings from gambling research, where event frequency is considered one of the strongest predictors of addictive behaviours (32, 33). Recent reviews further highlight that loot-box openings and pay-to-win mechanics show similar associations with problem gambling and gaming, again underscoring payment frequency as a central variable in the development of problematic gambling and gaming (31).

### 1.3 Dark patterns in in-game purchases

These structural similarities extend to the exploitation of players’ cognitive biases, often conceptualised as dark patterns (34). Developers employ strategies such as artificial scarcity, anchoring, and the manipulation of virtual currency exchange rates to sustain user engagement and spending (35). While specific mechanics such as content being available for limited amounts of time may enhance excitement and perceived enjoyment (28), recurring patterns designed to foster habitual play are frequently experienced as intrusive or burdensome. For instance, daily rewards that grant bonuses upon logging in are often compared to labour tasks, yet players still strive to collect them regularly (36).

Beyond visible design features, gaming operators may also employ data-driven personalization to adjust in-game systems dynamically. Although difficult to verify empirically, multiple industry patents describe mechanisms that can alter loot box probabilities, adjust item prices according to prior spending, or manipulate matchmaking to heighten the perceived desirability of specific cosmetic items (37).

Additional reinforcement mechanisms such as the battle pass, a one-time-payment that allows players to obtain in-game-items through continuous playing, further tie monetization to players’ psychological needs for competence and relatedness (38) offering a sense of continuous progress within social contexts for a recurring fee (39). Social dynamics themselves also shape spending patterns because studies show that peer purchasing frequency predicts higher in-game expenditure (40).

### 1.4 Expenditure concentrations

In 2018, more than 67% of the revenue in the mobile games sector was generated by just 5.5% of players (41). Across Europe, high spending of more than €80 per month is limited to 4% of adolescents (3). Players who spend disproportionately high amounts of money are referred to as ‘whales’, a term borrowed from the gambling industry and are highly valued by providers (42). These ‘whales’ represent about 10% of all players (43, 44). The definition of ‘whales’ varies across studies, ranging from self-perceived heavy spenders (45) to objectively identified top spending groups. Similar expenditure concentrations of high expenditure among a small proportion of players have been observed in traditional gambling (41, 46).

Little is currently known about individuals who spend large amounts of money in games, with one study suggesting that players of free-to-play games tend to be generally younger, male, and have migration backgrounds (43). Additionally, several surveys indicate a higher prevalence of gaming disorder among those who spend more money (11, 44, 47–49).

### 1.5 Regulation of loot boxes

The similarities between in-game purchases, particularly loot boxes, and gambling have sparked debates in both politics and academia. While the gaming industry has downplayed these concerns by comparing loot boxes to *Kinder Surprise* chocolate eggs (50), its strategy of self-regulation is widely regarded as inadequate. The disclosure of winning probabilities is often considered too opaque, and existing age-rating systems by PEGI (Pan-European Game Information, a Europe-wide age classification system for video games) and USK (Germany’s equivalent to PEGI) are deemed ineffective (51, 52).

Several European countries have therefore attempted to follow researchers’ calls for appropriate regulation (14). Among them are the Netherlands, which, after lengthy negotiations, are planning to introduce a ban on loot boxes (53). The model for this is Belgium, where the Gambling Commission classified loot boxes as a form of gambling, with violations punishable by fines of up to €800,000 (54). However, this Belgian ban appears to have had limited effect, as over 80% of the highest-grossing apps in Belgium still offer loot boxes (52).

In Austria, several consumer lawsuits have been filed against providers of loot boxes. At the time of writing, three court rulings have been issued in favour of the plaintiffs (55, 56). The reasoning for these decisions cited the legal definition of gambling, which also encompasses loot boxes, thereby requiring a license for their sale. The growing public and legal attention to the issue may partly explain why developers are increasingly removing loot boxes from their games. For example, in popular video games such as *Brawl Stars* and *Overwatch 2*, the purchase of loot boxes is no longer possible, although other dark patterns remain in place.

### 1.6 Research questions

Because adolescents’ impulse control is still developing and they constitute a key target group for digital game developers, questions arise concerning the extent of their financial engagement with digital games. The observed concentration of expenditure among a small number of players further raises the question, whether, as critically noted by scholars (30), vulnerable groups such as younger players or those affected by gaming disorder might be particularly at risk. It has also been assumed that economically disadvantaged individuals are more likely to be spending money in-game, as is the case in gambling (57, 58). Therefore, the present study focused on heavy spenders, examining their economic status and potential pathological behaviours. A crucial aspect of the research is the largely unresolved question of whether this small but high-revenue segment of players possesses comprehensive financial means or if these financing models disproportionately appeal to vulnerable, in this case financially disadvantaged, population groups. Therefore, the main research objectives were to: (i) investigate the extent of monetary expenditure on in-game purchases among adolescents in Austria, (ii) investigate influencing factors on the extent of monetary expenditure (gender, age, gaming disorder, gambling disorder), and (iii) examine the top 10% of paying players (heavy spenders [‘whales’]) who recorded the highest expenditures and compare them to casual spenders regarding socioeconomic status (SES) and gaming and gambling disorders.

Based on findings suggesting that digital games are a male-dominated medium and that males tend to spend higher amounts of money on digital games (59, 60), it was hypothesised that males would spend more money than females (H_1_). Age also seems to be a relevant factor because previous research indicates that high spenders in adulthood are predominantly aged between 25 and 49 years old (58). As financial resources generally increase with age, the potential to invest money in games is likely to rise accordingly. Therefore, it was hypothesised that the amount of money spent on in-game purchases would increase with age (H_2_). Spending on specific game mechanics, such as loot boxes, has also been associated with higher rates of problematic behaviour (16, 59). Similar to patterns observed in gambling, these findings suggest that greater monetary expenditure may be associated with problematic gaming behaviour. Therefore, it was hypothesised that gamers who spent more money on in-game purchases were more likely to exhibit symptoms of gaming disorder (H_3a_) and gambling disorder (H_3b_).

Previous research has indicated an unequal distribution of spending not only in gambling (45) but also in mobile games, where a small number of players account for a disproportionately large share of total revenue (41). Because mobile games have become the most popular type of games among adolescents, it was hypothesised there would be an unequal distribution of spending in the sample (H_4_). According to Lovell (44) approximately 10% of players qualify as heavy spenders (‘whales’). However, it remains unclear whether these heavy spenders are individuals with higher economic means, able to spend more due to greater financial resources, or whether players from economically disadvantaged backgrounds also spend large amounts of money on in-game purchases.

It was hypothesised that the latter would be the case (there would be no significant difference in economic status between heavy spenders and casual spenders; H_5a_). Moreover, it was hypothesised that heavy spending would be more frequently associated with gaming disorder (H_5b_) and gambling disorders (H_5c_) than casual spending.

## 2 Methods

### 2.1 Participants and procedure

The present study was based on the surveys conducted in Austria as part of the ‘Insert Coin to Continue’ study. For data collection, school classes across all Austrian federal states were chosen proportionately according to school year, school type, and federal state, with a total of 2,610 pupils being surveyed (61). This included the following types of schools: secondary schools (students aged between 10 and 14 years), polytechnic schools (students aged between 14 and 16 years), vocational schools (students aged between 15 and 19 years), vocational secondary schools (students aged between 14 and 19 years) and grammar schools (students aged between 14 and 18 years).

Based on these quotas, school classes were randomly selected from a complete list of Austrian schools using an Excel-based random-number procedure and school principals were contacted by the research team. Upon agreement, school administrations were provided with detailed study information, consent materials for parents or legal guardians, and standardised instructions for conducting the survey. Data collection was carried out by the schools themselves. Participating teachers were asked to administer the survey in the specified classes using a standardised online survey, which was accessed via a QR code or web link. Students completed the survey during class time using school-provided or personal digital devices. Participation was voluntary and anonymous.

A total of 2,610 students participated in the survey. During data cleaning, 302 cases were excluded because they were incomplete or did not meet the plausibility criteria for inclusion in the analytic dataset. These criteria concerned missing core variables, eligibility for the target age range, inconsistent response patterns, and implausible expenditure values. Regarding expenditure, high values were not excluded automatically, because previous research and media reports document exceptional cases of high adolescent spending on in-game purchases. However, expenditure values were excluded when they exceeded the predefined plausibility threshold of €15,000 over the past 12 months or appeared inconsistent with the overall response pattern. After exclusions, the final analytic sample comprised 2,308 participants. Of these, 1,952 participants reported having played games that included in-game purchases, 818 reported spending money on in-game purchases during the past 12 months, and 103 reported gambling for real money during the past 12 months and therefore completed the Brief Adolescent Gambling Screen (BAGS).

To ensure representativeness regarding school types, the distribution of the surveyed pupils was compared with the 2021/22 school statistics from Statistics Austria. The comparison showed that slightly more pupils in the sample attended secondary schools and polytechnic schools, the latter offering 1 year of direct vocational preparation. In contrast, students who attended a vocational school or a vocational middle school were underrepresented in the sample. The rates for attending a vocational or general secondary school corresponded with the data from Statistics Austria (62).

### 2.2 Measures

#### 2.2.1 Demographic measures

The survey collected sociodemographic information including age, gender, school type, school grade, federal state, and socioeconomic status. Socioeconomic status was assessed using the Family Affluence Scale III. Validated German translations of the Family Affluence Scale (FAS) and the Gaming Disorder Test (GDT) were used. The BAGS was translated into German for the present study.

#### 2.2.2 Family Affluence Scale (FAS III)

The six-item FAS III (63) was used to assess the economic status of families via indirect questions. The items revolve around the housing situation, vacations, and luxury products in the family, which are combined into a sum score. A sample item is: “Does your family own a car, van or truck?” Responses are scored according to the number of vehicles owned (*no*, *yes, one*, *yes, two or more*), with higher scores reflecting higher levels of family affluence. The validity of the FAS as an instrument for surveying income has been verified (63–66).

#### 2.2.3 Gaming Disorder Test

Gaming disorder was assessed using the four-item Gaming Disorder Test (67). The scale assesses core symptoms of gaming disorder and combines the items into a sum score.

A sample item is: *“I have had difficulties controlling my gaming activity.”* Responses are rated on a five-point Likert scale ranging from 1 (*never*) to 5 (*very often*), with higher scores indicating a greater likelihood of gaming disorder. Sum scores range from 4 to 20, and a cut-off score above 15 was used to indicate probable gaming disorder. In the present study, the GDT demonstrated acceptable internal consistency (Cronbach’s *α* = 0.768).

#### 2.2.4 Brief Adolescent Gambling Screen

Gambling disorder was assessed using the three-item Brief Adolescent Gambling Screen (68). The items assess key indicators of gambling disorder and are combined into a sum score.

A sample item is: *“How often have you hidden your gambling/betting from your parents, other family members or teachers?”* Responses are scored on a four-point scale from 0 (*Never*) to 3 (*All of the time*) and summed to yield a total score ranging from 0 to 9, with higher scores indicating a higher likelihood of gambling disorder. Scores of 0–3 indicate a very low probability of gambling disorder, scores of 4–5 indicate a probable gambling disorder, and scores of 6 or higher indicate a very high probability. In the present study, the BAGS showed acceptable internal consistency (Cronbach’s *α* = 0.70). Only participants who reported gambling within the past 12 months (*n* = 103) completed this measure. Because no validated German version of the BAGS was available, the original English items were translated into German by a language professional and reviewed by the research team for semantic equivalence and age-appropriate wording. The German version should therefore be understood as an adapted screening instrument rather than a psychometrically validated German scale.

#### 2.2.5 Gini coefficient

The Gini coefficient is a statistical measure used to quantify inequalities within a group (69). It is often applied to measure income differences and to assess disparities in money expenditure (40, 45) and has found applications in numerous scientific disciplines (70). The coefficient ranges from 0 to 1, with a value close to 0 indicating that each member of the group earns the same amount, and a value close to 1 indicating that one member has all the income.

### 2.3 Ethical considerations

The study was conducted in accordance with established ethical guidelines and principles. The study protocol and data collection procedures were reviewed and approved by the ethics committees of the Austrian educational authorities. Prior to data collection, detailed information about the study was provided to school administrations, teachers, parents or legal guardians, and students. Parents and legal guardians received study information beforehand and could decline participation; student assent was obtained. Participation was voluntary and anonymous. No personally identifiable data were collected. Data were processed and stored in compliance with applicable data protection regulations.

### 2.4 Data analysis

Data analyses were conducted using *IBM SPSS Statistics (Version 24)*. Prior to analysis, data were screened for completeness and plausibility. Surveys with incomplete or implausible responses were excluded, resulting in a final analytical sample of 2,308 participants. Analyses focusing on in-game spending were conducted using the subsample of respondents who reported having spent money on in-game purchases within the past 12 months (*n* = 818). Analyses related to gambling disorder were restricted to participants who reported gambling during the same period (*n* = 103). Descriptive statistics for sociodemographic characteristics, gaming behaviour, and in-game expenditure were calculated. Due to the skewed distribution of expenditure data, non-parametric statistical tests were applied where appropriate. Group differences between two independent groups were examined using Mann–Whitney U tests, while differences across multiple age groups were analysed using Kruskal–Wallis tests with *post-hoc* comparisons. Associations between continuous variables were assessed using Spearman rank correlations. To reduce reliance on categorical screening cut-offs, analyses involving gaming disorder and gambling disorder were supplemented by continuous-score analyses using the GDT and BAGS sum scores. Specifically, continuous scores were correlated with in-game expenditure, and heavy and casual spenders were compared on continuous GDT and BAGS scores using Mann–Whitney U tests.

Gender differences and associations between categorical variables, including spending groups and the prevalence of gaming and gambling disorder, were examined using chi-square tests. Effect sizes are reported where appropriate. Inequality in in-game expenditure was assessed using the Gini coefficient and visualised with a Lorenz curve. To identify heavy spenders (“whales”), participants in the top 10% of total in-game expenditure over the past 12 months were classified as heavy spenders and compared with casual spenders regarding socioeconomic status and gaming and gambling disorder.

To control for potential confounding, a multivariable binary logistic regression was conducted with heavy-spender status as the dependent variable and age, gender, FAS III sum score, and GDT sum score as predictors. Due to the small number of non-binary participants, gender was coded as a binary variable for this analysis and non-binary participants were excluded from the regression model.

## 3 Results

### 3.1 Sample description

Table 1 shows the distribution of the variables sex and age for the overall sample, along with screening results for gaming and gambling disorders among participants that had made in-game purchases in the past 12 months. Of the total sample, 1952 participants (84.6%) had played games that allow in-game-purchases (92.5% male, 76.7% female). A total of 818 participants (35.4%) reported spending money in games in the past year. Of these 818 participants, 103 (12.6%) indicated that they had participated in gambling for real money (slots, casino, online gambling). The gender distribution in the sample was nearly balanced, with a relatively even distribution between the four age groups.

**Table 1:** Distribution of variables sex and age.

Overall sample (*N* = 2,308)		Participants who made in-game purchases in the past 12 months (*n* = 818)	
Variables	n (%)	Variables	n (%)
Male	1,126 (48.8%)	Male	615 (75.2%)
Female	1,150 (49.8%)	Female	188 (23.0%)
Non-binary	32 (1.4%)	Non-binary	15 (1.8%)
Total	2,308 (100%)	Total	818 (100%)
10–12 years	567 (24.6%)	10–12 years	199 (24.3%)
13–14 years	660 (28.6%)	13–14 years	246 (30.1%)
15–16 years	675 (29.2%)	15–16 years	248 (30.3%)
17–19 years	406 (17.6%)	17–19 years	125 (15.3%)
Total	2,308 (100%)	Total	818 (100%)
		Gaming disorder	43 (5.3%)
		Gambling disorder^1^	32 (3.9%)

1 Gambling disorder was assessed only participants who completed the BAGS (*n* = 103).

### 3.2 Demographics

As aforementioned, a total of 1952 of the sample (84.6%) had played a game that included in-game purchases, with male participants using them more frequently (92.5% male, 76.7% female). In the past 12 months, 818 of the total sample (35.4%) reported having spent money in digital games, corresponding to 41.9% of those who played videogames that featured in-game-purchases. When asked about the payment methods used for in-game purchases, participants most frequently mentioned prepaid cards such as the *Google Play Card* or *Paysafe* cards (63.8%), followed by *PayPal* (23.0%), their parents’ credit or debit card (17.6%), their own debit card (16.8%), buy-now-pay-later services such as *Klarna* (5.3%), and billing through their mobile phone contract (2.0%). Among the adolescents who provided information on their financial resources (*n* = 811), the average amount of money available per month was €149.47 (median = €50; SD = €299.87). These funds stemmed primarily from pocket money, monetary gifts for birthdays or report cards, and from small paid tasks for relatives.

Statistical analysis showed pronounced gender differences among those who made in-game-purchases (75.2% male, 23.0% female, 1.8% non-binary; χ^2^, *p* < 0.001; *n* = 818). The age distribution of purchasers was similar to that of the total sample. Among past-year in-game spenders, 5.3% met criteria for gaming disorder. Probable gambling disorder was identified in 32 participants, corresponding to 3.9% of all past-year in-game spenders and 31.1% within the BAGS subsample (*n* = 103). Because the BAGS was administered only to participants who reported past-year gambling, gambling disorder prevalence should be interpreted with reference to this subgroup. Regarding migration background, 30.8% of participants who spent money in games in the past 12 months had both parents born outside of Austria (Table 2).

**Table 2:** Distribution of expenditure on in-game purchases (*n* = 818 adolescents who made in-game purchases in the past 12 months).

Variable	≤€49 n (%)	€50–199 n (%)	≥€200 n (%)	Total n (%)	Mean
Female	101 (53.7%)	61 (32.5%)	26 (13.8%)	188 (100%)	101.8€
Male	237 (38.5%)	242 (39.4%)	136 (22.1%)	615 (100%)	180.9€
10–12 years	101 (50.7%)	73 (36.7%)	25 (12.6%)	199 (100%)	104.4€
13–14 years	114 (46.3%)	92 (37.4%)	40 (16.3%)	246 (100%)	163.1€
15–16 years	80 (32.3%)	100 (40.3%)	68 (27.4%)	248 (100%)	215.6€
17–19 years	46 (36.8%)	44 (35.2%)	35 (28.0%)	125 (100%)	196.8€

### 3.3 Gender and age differences

On average, the adolescents surveyed spent €169.9 on in-game purchases in the past 12 months, which corresponds to a monthly expenditure of around €14.2. Boys reported higher spending (€180.9) compared to girls (€101.8). A Mann–Whitney U test indicated a significant gender difference regarding in-game purchases in the past 12 months (*p* < 0.001) with male participants spending significantly more money (M_Rank_ = 420.54, *n* = 615) than female participants (M_Rank_ = 341.36, *n* = 188): U = 46409.00, Z = −4.104, *p* < 0.001, *r* = −0.145.

Regarding age, it was assumed that the older participants got, the more money they would have at their disposal and therefore the more money to buy in-game items. However, the age group of 15- to 16-year-olds stood out with an average expenditure of over €200 per year. Due to the non-parametric distribution of expenditures, a Kruskal-Wallis test was performed to compare 10–12-year-olds (M_Rank_ = 366.33), 13–14-year-olds (M_Rank_ = 369.80), 15–16-year-olds (M_Rank_ = 443.86) and 17–19-year-olds (M_Rank_ = 435.06). The results were significant (*p* < 0.05), indicating age group differences. Post-hoc comparisons showed that participants aged 15–16 spent significantly more money than those in all other age groups. Rather than a linear age effect, these findings suggest a peak in spending during mid-adolescence.

### 3.4 Problematic engagement

It was examined whether the GDT sum score was associated with the amount of money spent on in-game purchases during the past 12 months. The Spearman coefficient showed a highly significant positive correlation of medium effect size with r_s_ = 0.257 (*p* < 0.001), indicating that a higher scale value and consequently a greater likelihood of gaming disorder being associated with higher money expenditure.

Using an additional Mann–Whitney-U test, it was shown that participants with gaming disorder (M_Rank_ = 549.27, *n* = 43) spent significantly more money on in-game purchases in the past 12 months than those without gaming disorder (M_Rank_ = 401.75, *n* = 775): U = 10,652,500, Z = −3.992, *p* < 0.001, *r* = 0.140. Although the effect size was small, the results nevertheless point to a meaningful association between problematic gaming tendencies and higher monetary engagement in digital games.

It was also examined whether there was a significant correlation between the sum score of the BAGS and the amount of money spent on in-game purchases in the past 12 months. The Spearman rank correlation showed a highly significant positive correlation of medium effect size (*r*_s_ = 0.321, *p* < 0.001), indicating that a greater likelihood of gambling disorder correlated with higher money expenditure. Given the restricted BAGS subsample and the non-validated German adaptation of the instrument, all findings involving gambling disorder should be considered exploratory screening findings rather than diagnostic prevalence estimates.

### 3.5 Distribution of in-game expenditure

**Figure 1:**
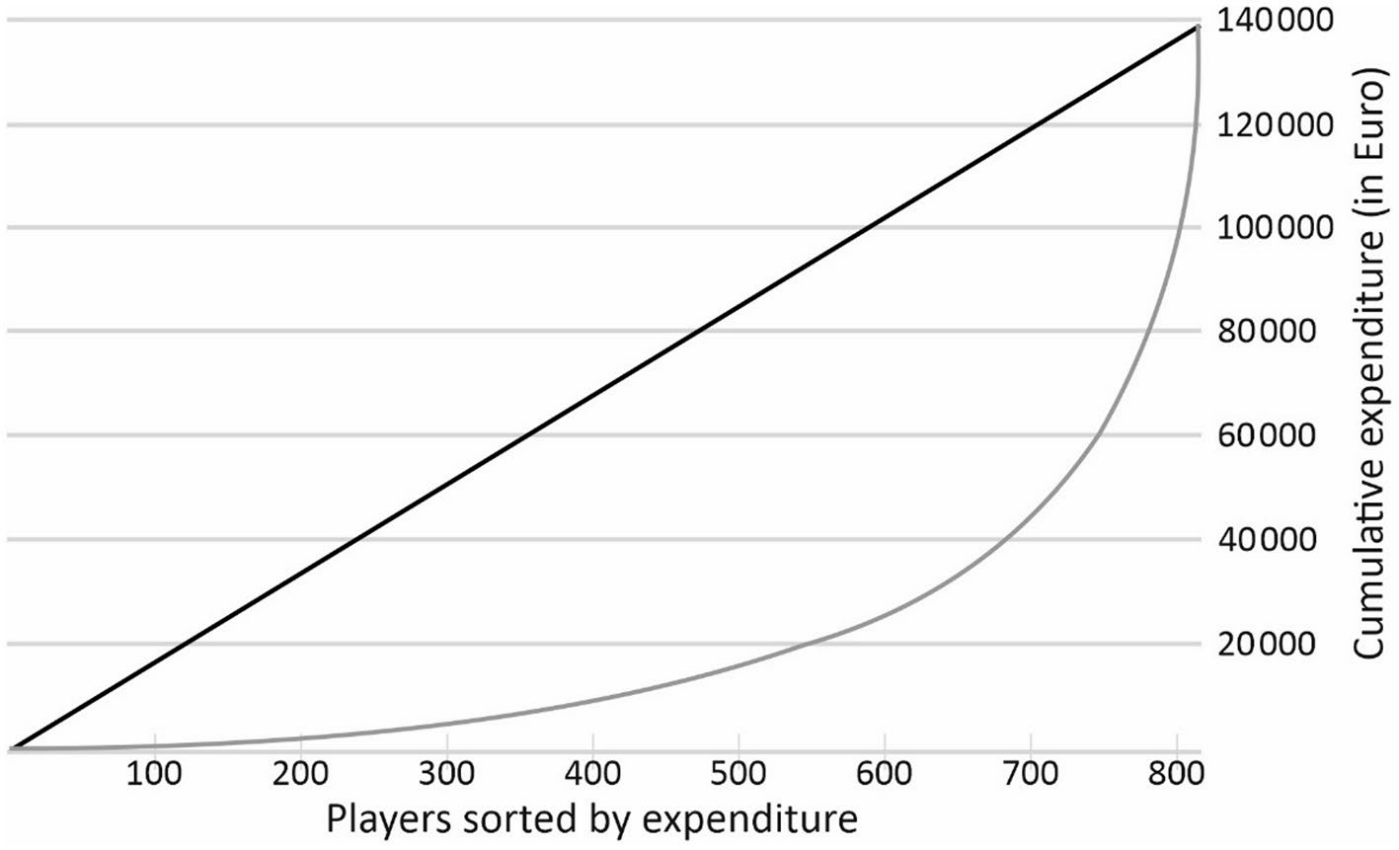
Distribution of cumulative expenditure on in-game purchases; *n* = 818 (adolescents who made in-game purchases in the past 12 months).

As can be seen in Figure 1, a large proportion of participants spent relatively little money, while a smaller group accounted for substantial expenditures. In the present study, the Gini coefficient for money spent on in-game purchases in the past 12 months was 0.854, which indicates a highly unequal distribution of monetary expenditure among individual participants.

The unequal distribution of expenditures among individual participants can be visualised using the Lorenz curve (Figure 1). The y-axis represents the cumulative expenditure of all participants over the past 12 months. The diagonal line illustrates perfect equality, where each paying individual contributes the same amount, while the curved line depicts the actual distribution in the sample, showing that spending was concentrated among a small number of players. It is evident that most participants accounted for only a small proportion of the cumulative expenditure, while the majority of expenditure was generated by a small subset of participants. In the sample, 10% of paying players (heavy spenders) were responsible for 61.4% of total in-game expenditures (€85,385.9) during the past 12 months.

To explore parallels between gambling and gaming, the proportion of in-game expenditure attributable to participants with pathological behaviours was analysed. Over the past 12 months, the participants in the sample (*n* = 818) collectively spent €138,952.5 on in-game purchases. Participants meeting the criteria for gaming disorder (*n* = 43; 5.3%) accounted for 10.3% of this expenditure, while those identified with gambling disorder (*n* = 32; 3.9%) accounted for 14.7% of the expenditure. The 3.9% figure refers to all past-year in-game spenders; within the BAGS subsample of past-year gamblers (*n* = 103), 31.1% met criteria for probable gambling disorder.

### 3.6 Heavy in-game spenders

Given the strong concentration of spending, it was essential to examine who the heavy spenders were and what characteristics distinguish them. The participants who reported spending between €400 and €12,000 on in-game purchases during the 12 months preceding the survey (n = 85; 10.4% of participants; M = €1.005) were classified as heavy spenders.

A comparison of heavy spenders and casual spenders (Table 3) shows heavy spenders were predominantly male and aged 15 to 16 years. The average age of heavy spenders was 15.04 years, whereas all 818 participants who spent money on in-game purchases in the past 12 months had an average age of 14.26 years. Therefore, heavy spenders in the sample were older than casual spenders. Among female participants, 5.3% were heavy spenders, compared to 11.4% of male participants, indicating a significantly higher proportion of males as heavy spenders (χ^2^(1) = 5.901, *p* = 0.015, *n* = 803).

**Table 3:** Distribution of the variables gender, age and gaming disorder for casual and heavy spenders.

Casual spenders (*n* = 733)		Heavy spenders (*n* = 85)	
Variables	n (%)	Variables	n (%)
Male	545 (74.3%)	Male	70 (82.3%)
Female	178 (24.3%)	Female	10 (11.8%)
Non-binary	10 (1.4%)	Non-binary	5 (5.9%)
Total	733 (100%)	Total	85 (100%)
10–12 years	188 (25.6%)	10–12 years	11 (12.9%)
13–14 years	226 (30.8%)	13–14 years	20 (23.5%)
15–16 years	213 (29.1%)	15–16 years	35 (41.2%)
17–19 years	106 (14.5%)	17–19 years	19 (22.4%)
Total	733 (100%)	Total	85 (100%)
Gaming disorder	31 (4.2%)	Gaming Disorder	12 (14.1%)

Economic status was assessed using the FAS III, which yields a total score ranging from 0 to 13 points. Based on the collected summed values, participants were categorised into low (0–7 points), medium (8–11 points), and high economic status (12–13 points). The mean FAS III score of the heavy spenders (9.58) did not differ significantly from that of casual spenders (9.71; Table 4). The Mann–Whitney U test confirmed no significant difference in the summed score of the FAS III between heavy spenders (M_Rank_ = 417.15, *n* = 85) and casual spenders (M_Rank_ = 408.61, *n* = 733), U = 30502.000, Z = − 0.319, *p* = 0.750.

**Table 4:** Distribution of economic status according to FAS III and migration background.

Casual spenders (*n* = 733)		Heavy spenders (*n* = 85)	
Variables	n (%)	Variables	n (%)
Low ES	189 (25.6%)	Low ES	22 (25.9%)
Medium ES	400 (54.6%)	Medium ES	45 (52.9%)
High ES	144 (19.6%)	High ES	18 (21.2%)
Total	733 (100%)	Total	85 (100%)
Migration background	224 (30.6%)	Migration background	28 (32.9%)

The prevalence of gaming and gambling disorders was higher among heavy spenders than among casual spenders (Table 3). Because neither group met the assumption of normal distribution (Shapiro–Wilk *p* < 0.001), a Mann–Whitney U test was conducted to calculate differences in the summed score of the GDT. There was a significant difference regarding the summed score of the GDT, with heavy spenders (M_Rank_ = 512.50, *n* = 85) having a higher value than the casual spenders (M_Rank_ = 397.56, n = 733): U = 22397.500, Z = −4.270, *p* < 0.001, *r* = 0.149 (representing a small effect size). In addition, a chi-square test confirmed a significant association between heavy spending and the prevalence of gaming disorder: χ^2^(1) = 14.954, *p* < 0.001, *n* = 818.

To examine differences in gambling disorder, a Mann–Whitney U test was performed on BAGS summed scores. Heavy spenders (M_Rank_ = 72.31, *n* = 24) had significantly higher values than casual spenders (M_Rank_ = 45.83, n = 79): U = 460.500, Z = −3.865, *p* < 0.001, *r* = 0.243. A chi-square test also confirmed a significant association between heavy spending and gambling disorder: χ^2^(1) = 14.44, *p* < 0.001, *n* = 103. Because the BAGS was completed only by past-year gamblers, analyses involving gambling disorder were restricted to the BAGS subsample (*n* = 103). Within this subsample heavy spenders showed higher BAGS scores and a higher prevalence of probable gambling disorder.

A multivariable binary logistic regression was conducted with heavy-spender status as the dependent variable and age, gender, FAS III sum score, and GDT sum score as predictors (see Table 5). Due to the small number of non-binary participants, gender was coded as a binary variable and non-binary participants were excluded from this analysis (n = 803). BAGS scores were not included in the regression model because they were only available for the small subsample of participants who reported real-money gambling. Age (OR = 1.22, 95% CI [1.08, 1.37], *p* < 0.001), male gender (OR = 2.14, 95% CI [1.07, 4.29], *p* = 0.032), and GDT sum score (OR = 1.13, 95% CI [1.06, 1.19], *p* < 0.001) significantly predicted heavy-spender status, whereas FAS III sum score did not (OR = 1.08, 95% CI [0.96, 1.21], *p* = 0.220). The model explained a modest proportion of variance in heavy-spender status (Nagelkerke R^2^ = 0.082) and model fit was acceptable (Hosmer–Lemeshow test: χ^2^(8) = 10.42, *p* = 0.237). Therefore, gaming disorder symptoms remained associated with heavy-spender status after adjustment for demographic and socioeconomic variables, although the modest Nagelkerke R^2^ indicated that heavy-spender status was only partly explained by the included predictors.

**Table 5:** Binary logistic regression predicting heavy-spender status (*n* = 803 male and female adolescents who made in-game purchases in the past 12 months).

Predictor	B	SE	Wald	df	*p*	OR	95% CI Lower	95% CI Upper
Gender (male)	0.760	0.355	4.58	1	0.032	2.14	1.07	4.29
GDT sum score	0.119	0.030	15.42	1	<0.001	1.13	1.06	1.19
Age	0.198	0.060	10.92	1	<0.001	1.22	1.08	1.37
FAS III sum score	0.072	0.059	1.50	1	0.220	1.08	0.96	1.21

It is also noteworthy that the proportion of participants with a migration background was comparatively high among those who reported spending money in-game. To examine this relationship, potential associations between migration background, spending level, and socioeconomic status were analysed. A chi-square test showed no significant association between migration experience and spending group (*χ^2^*(1) = 0.20, *p* = 0.65, *n* = 818), suggesting that overall spending patterns did not differ significantly based on migration experience. Moreover, a Mann–Whitney U test showed that participants with migration experience (M_Rank_ = 351.09) scored significantly lower on the FAS III than those without migration experience (M_Rank_ = 435.51): U = 56,597.00, Z = −4.77, *p* < 0.001, indicating lower financial security.

Sensitivity analyses using alternative top 5% and top 15% thresholds showed the same substantive pattern as the primary top 10% definition. Across all thresholds, GDT sum score remained a significant predictor of heavy-spender status, whereas FAS III sum score did not significantly predict heavy-spender status. Age and gender were less stable in the more restrictive top 5% model, likely reflecting the smaller number of heavy spenders and reduced statistical power.

## 4 Discussion

### 4.1 Gender and age aspects of in-game spending

The present study’s findings confirmed in-game-purchases as a routine part of some adolescents’ gaming behaviour, with clear gender differences both in participation and spending. This difference likely reflects broader patterns of digital play, because videogames are more commonly played by boys and play a greater role in male socialisation (71, 72). Male participants also spent significantly more on in-game purchases than their female counterparts, supporting (H_1_). This may be due to several factors. For instance, male socialisation is often more oriented towards competition, and competitive motives are more prevalent among male gamers. Previous research has also shown competition and achievement as key motivational factors among male players (71).

In the context of pay-to-win mechanics, such achievement-oriented motivation may translate into higher in-game spending because players invest money to gain competitive advantages or status-related rewards. Because spending money in some games can provide gameplay advantages, it may result in higher expenditure among males. Moreover, studies indicate that males spend more time playing digital games compared to females (72, 73), which increases the opportunity for spending. Additionally, the exclusion of women from gaming environments, where hate speech and discrimination are prevalent (74–76), may further contribute to their lower in-game spending.

Contrary to H_2_, which assumed a linear increase in spending with age, expenditure peaked among 15- to 16-year-olds and declined thereafter, indicating that financial engagement with videogames follows a developmental curve rather than a steady increase with age. This period likely reflects the phase when gaming is most central to peer culture and identity formation. Therefore, adolescent spending cannot be explained solely by disposable income, but also by the symbolic and social significance of gaming within youth culture (72), before declining again as older adolescents shift priorities towards relationships, work and/or other leisure activities.

Adolescents with a migration background were overrepresented among those who spent money in-game (30.6% of all spenders and 32.9% of heavy spenders), compared to 21.4% in the general population (77). Despite lower average financial security, this group participated in spending at comparable levels. Therefore, gaming may provide symbolic inclusion and belonging for socially marginalised youth, while simultaneously exposing them to heightened risks of financial over-engagement.

### 4.2 In-game-spending and addictive behaviours

The present study’s findings showed significant associations between spending intensity and symptoms of gaming disorder and gambling disorder (supporting H_3a_ and H_3b_). This is consistent with prior research associating different forms of in-game purchases to problematic behaviours (7, 11, 17, 58). Higher spending was associated with elevated scores on both the GDT and the BAGS, indicating that the intensity of in-game spending was associated with problematic engagement. However, because BAGS analyses were restricted to the relatively small subsample of adolescents who reported gambling in the past 12 months, findings related to gambling disorder should be interpreted with caution. While causality cannot be inferred, two interpretations seem plausible: spending money in-game may contribute to the development of problematic behaviours, or individuals who already display such behaviours may be more inclined to spend excessively in-game. In either case, these results imply that a substantial share of in-game revenues may stem from vulnerable individuals who are particularly susceptible to problematic engagement. Importantly, the present study examined all types of in-game spending. Therefore, the findings suggest that debates about gaming–gambling convergence should not be limited to randomised mechanics such as loot boxes but should also consider broader patterns of in-game spending, while recognising that the present study assessed aggregate expenditure rather than specific monetization mechanics.

These findings should be interpreted with careful attention to the conceptual distinction between gaming disorder and gambling disorder. The present study measured overall in-game expenditure and did not distinguish between specific chance-based rewards, competitive advantages, cosmetic items, or other forms of monetized content. Moreover, analyses involving gambling disorder were based on the relatively small subsample of adolescents who reported gambling in the past 12 months. Accordingly, the associations observed for gaming disorder and gambling disorder should not be understood as interchangeable, and findings related to gambling disorder should be interpreted with caution.

### 4.3 Spending disparities and convergence with gambling

The Gini coefficient for in-game expenditure in the present sample (0.854) indicated a pronounced concentration of spending, comparable to levels reported for gambling markets (41, 46). This highly unequal distribution empirically supports claims that free-to-play monetization models rely on a small subset of high-spending users who generate a disproportionate share of total revenue (42). Accordingly, H_4_, which assumed an unequal distribution of in-game spending within the sample, was supported.

Expenditure patterns are often described using the Pareto principle, which assumes that approximately 20% of consumers account for 80% of total spending, corresponding to a Gini coefficient of around 0.6 (76). However, gambling expenditures have been shown to exceed this level of concentration. In Germany, gambling-related spending have been reported to have a Gini coefficient of 0.879, indicating an extreme concentration of expenditure among very few individuals (40, 45).

Given that in-game purchases are presented as optional and frequently serve status-related or competitive functions comparable to those of luxury goods, a similarly unequal distribution of spending might be expected. The observed Gini coefficient of 0.854 for in-game purchases in the past 12 months closely mirrors the concentration reported for gambling expenditures in Germany, suggesting structural similarities between the two markets.

Moreover, previous research indicates that a substantial proportion of gambling industry revenue originates from individuals with gambling disorders, with estimates of approximately 32% in Germany (40, 45). The observed parallels in expenditure concentration are consistent with possible structural similarities between in-game purchasing and gambling markets. However, because the present study used cross-sectional self-report data and assessed aggregate expenditure rather than specific monetization mechanics, these findings should be interpreted as hypothesis-generating rather than as evidence of convergence or causal harm mechanisms.

Reinforcement mechanisms known from gambling, such as the illusion of control, loss aversion, and the near-miss effect (24–26, 34), are embedded within monetization systems of digital games and have been conceptualised as dark patterns that exploit cognitive biases. By encouraging repeated purchases under conditions of uncertainty or urgency, these systems reproduce spending dynamics typically associated with gambling, known to carry substantial risks for problematic engagement, particularly among adolescents (78).

The study’s findings demonstrate that the intensity of in-game spending, independent of specific mechanics, correlates with scores on both gaming and gambling disorder scales. This aligns with previous research showing comparable associations across diverse monetization formats (11, 47, 48). However, potential harm might not be confined to particular features but may also be rooted in the broader incentive structures of digital game financing models.

### 4.4 Heavy spenders (whales)

The concentration of in-game expenditure among the top 10% of players, responsible for over 60% of total spending, underscores the existence of a small but economically crucial segment of heavy spenders (i.e., whales) (43). In the present study, the top 10% of players (heavy spenders) accounted for 61.4% of all in-game spending. Consistent with previous research (43), these players were predominantly male, slightly older, and significantly more likely to meet criteria for gaming disorder and gambling disorder. In the sample, results from the Family Affluence Scale (FAS III) further showed that economic status did not significantly influence spending behaviour, meaning that adolescents from lower-income families spent roughly the same absolute amounts of money as their more affluent peers. Therefore, H_5a_ was supported because no significant differences in socioeconomic status were observed between heavy spenders and casual spenders. This finding suggests that high in-game expenditure among adolescents cannot be explained solely by greater material resources.

Gambling research has shown a similar pattern, where high expenditure was found across all income groups, yet its impact was disproportionately greater for financially and socially disadvantaged individuals, who spend a higher share of their available resources (57, 58). While this means that no social group is inherently protected from overspending, it also implies that the consequences of high expenditure are not evenly distributed. Adolescents with fewer economic or social resources may experience greater harm from the same level of spending, simply because they possess fewer financial and social buffers to absorb the loss. In this respect, the relevant question is not only whether socioeconomic status predicts heavy spending, but whether comparable levels of spending may have unequal consequences across socioeconomic groups. Adolescents with fewer financial resources may experience the same absolute expenditure as a greater relative burden, even if they are not more likely to spend heavily. Therefore, protecting these groups requires shifting responsibility away from individual self-control and towards regulatory oversight of design practices that generate disproportionate financial pressure on players.

The findings also showed that adolescents with a migration background were slightly overrepresented among heavy spenders despite lower average family affluence. This also aligns with previous research (43) reporting higher gaming expenditures among players with migration backgrounds, suggesting that social marginalisation may heighten susceptibility to the affective and social gratifications offered by free-to-play monetization models. In this sense, heavy spenders represent a doubly vulnerable group, psychologically due to higher rates of addictive symptoms, and socially due to limited financial or structural resources.

Both descriptive analyses and hypothesis testing showed an association between in-game spending and problematic gaming, with the prevalence of gaming disorder among heavy spenders (14.1%) being significantly higher than among casual spenders (4.2%). Total in-game expenditure also correlated positively with GDT scores, indicating that higher in-game spending is associated with problematic gaming engagement. Adolescents identified as having a gaming disorder spent significantly more money than their peers without such symptoms, underscoring an association between problematic gaming and excessive monetary engagement. These findings support H_5b_, indicating that heavy spending was associated with a higher prevalence of gaming disorder symptoms. This association remained significant in the multivariable regression model after adjustment for age, gender, and socioeconomic status. At the same time, the modest Nagelkerke R^2^ value indicates that heavy-spender status might be influenced by additional factors not included in the model, such as gaming intensity, peer norms, exposure to persuasive monetization features and dark patterns or access to payment methods. Because of the cross-sectional design no conclusions can be drawn about causality or directionality.

This pattern is consistent with previous research showing that a substantial proportion of in-game revenue is generated by users displaying problematic gaming engagement (41, 79). At the same time, higher spending should not be interpreted exclusively as an indicator of problematic behaviour. Prior studies show that in-game spending may also reflect non-pathological motivations, including enjoyment, personalization, social identity, status display, competitive aspirations, or perceived progression within the game (80). These motivations may be particularly salient during adolescence, when peer recognition and identity formation are developmentally important. Nevertheless, the present findings indicate that a meaningful share of total in-game expenditure was reported by players showing symptoms of behavioural addiction. This raises concerns that current monetization systems may be disproportionately used by adolescents reporting symptoms of problematic use, although the cross-sectional design precludes conclusions about directionality or causality.

In addition, H_5c_ was supported. Within the BAGS subsample of past-year gamblers, heavy spenders were more likely than casual spenders to meet criteria for probable gambling disorder. Although this finding should be interpreted with caution due to the relatively small number of participants reporting gambling behaviour, it is consistent with previous research indicating an overlap between excessive spending patterns and gambling-related problems (21, 79).

### 4.5 Implications for research and policy

The present findings identify heavy spenders as a subgroup of adolescent players who may warrant closer attention in future research and consumer-protection debates concerning free-to-play monetization. Heavy spenders were predominantly male, concentrated in mid-adolescence, and reported higher levels of gaming disorder symptoms than casual spenders. Because this relatively small group accounted for a disproportionate share of total expenditure, the findings raise questions about how spending-related risks among minors can be better understood and addressed. However, the cross-sectional design of the present study precludes conclusions about whether in-game monetization practices contribute causally to problematic engagement or whether specific consumer-protection measures would reduce such risks.

The findings also suggest that the consequences of high in-game expenditure may differ across socioeconomic groups. Socioeconomic status did not predict heavy-spender status, and comparable absolute levels of spending may represent a greater relative burden for adolescents with fewer financial resources. Policy discussions should therefore consider in-game purchases beyond loot boxes. Existing regulatory efforts have primarily focused on loot boxes, yet their effectiveness and scope remain contested (14, 51, 52, 81). Future research should evaluate which measures, such as expenditure transparency tools, optional or age-appropriate spending limits, or restrictions on particularly persuasive monetization features (e.g., dark patterns in video games), may reduce potential harms without limiting legitimate gameplay. Such evidence-based policy development would benefit from independent access to industry transaction data, which would allow spending patterns and the effects of protective measures to be assessed more directly.

## 5 Limitations

The present study has several limitations. The findings of the study were based on self-reported expenditure data, which are subject to well-documented biases in recall and reporting accuracy. For instance, previous research has shown that gamblers tend to underestimate their spending (82) and that the degree of misreporting increases with higher levels of expenditure (83). Similar biases are likely present in the present study. Adolescents may underestimate or misremember the amount of money spent on in-game purchases, particularly when purchases are made repeatedly, through different payment methods, or in small amounts over time. This issue may be especially relevant for heavy spenders, for whom recall bias may be greater due to the higher number and frequency of transactions. These reporting biases may have influenced both prevalence estimates and the magnitude of the observed associations. To reduce potential distortions, responses indicating implausible spending amounts were excluded, with the upper plausibility threshold informed by media reports of exceptionally high adolescent spending on digital games (84–86). Second, although validated instruments were used to assess gaming disorder and gambling disorder, reporting biases cannot be ruled out for these self-reported measures. Moreover, the BAGS was translated into German but has not been validated in German. Analyses involving gambling disorder were also based on the relatively small subsample of adolescents who reported gambling in the past 12 months. Therefore, although significant associations were observed, these findings should be interpreted with caution because the small subsample limits statistical robustness. Consequently, BAGS-based prevalence estimates should not be generalised to the full sample of adolescent in-game spenders, and the use of established BAGS cut-offs in the German adaptation should be regarded as provisional. Third, the cross-sectional design precludes causal inferences regarding the directionality of the observed associations. Finally, the school-based, quota-based sampling approach limits the generalizability of the findings to adolescents outside formal education and to populations beyond the Austrian context and findings may not generalise to adolescents in countries with different gaming cultures, parental payment practices, consumer protection frameworks, gambling regulations, or levels of disposable income.

## 6 Conclusion

The present study’s findings show that adolescent in-game spending is highly concentrated among a small subset of players who also display elevated symptoms of gaming disorder and, within the subgroup assessed for gambling behaviour, gambling disorder. This concentration occurred independently of socioeconomic status, suggesting that high expenditure is not limited to adolescents with greater financial means. Heavy spending was associated with elevated symptoms of gaming disorder and gambling disorder, raising consumer protection concerns regarding potentially vulnerable high-spending users. The findings are consistent with concerns raised in the literature about “predatory monetization” models (30), although the present cross-sectional data cannot determine whether specific monetization practices contribute causally to problematic gaming behaviour (31). Results suggest that current consumer protection approaches may not fully address risks among adolescent players. Effective youth and consumer protection may therefore benefit from structural safeguards, such as spending limits, transparency requirements, and restrictions on persuasive monetization practices, to reduce the risk that digital in-game revenues are disproportionately derived from those most susceptible to harm.

## Data Availability

The raw data supporting the conclusions of this article will be made available by the authors, without undue reservation.
