# Supplementary material for: Heavy spenders in digital games: expenditure disparities and associations between microtransactions, gaming disorder and gambling disorder among adolescents
Source: Front Public Health. 2026 Aug 21;14:1867460. doi: 10.3389/fpubh.2026.1867460 (PMC13541447; doi:10.3389/fpubh.2026.1867460)
Supplement: Supplementary file 1 [file Table_1.DOCX]

**Supplementary Table 1** Sensitivity analyses using alternative heavy-spender thresholds

| Variables | Heavy-spender threshold | | |
| --- | --- | --- | --- |
|  | Top 5% (n = 42) | Top 10% (n = 85) | Top 15% (n = 130) |
| FAS III sum score | U = 15,570.50,  p = .623 | U = 30,502.00,  p = .750 | U = 41,959.50,  p = .259 |
| GDT sum score | U = 11,459.50,  p < .001 | U = 22,397.50,  p < .001 | U = 34,640.00,  p < .001 |
| Gaming disorder | χ²(1) = 15.432,  p < .001 | χ²(1) = 14.954,  p < .001 | χ²(1) = 11.724,  p < .001 |
| BAGS sum score | U = 129.50,  p < .001 | U = 460.50,  p < .001 | U = 701.50,  p = .002 |
| Gambling disorder | χ²(1) = 19.920,  p < .001 | χ²(1) = 14.440,  p < .001 | χ²(1) = 11.701,  p < .001 |
